# The association of self-reported awake bruxism with anxiety, depression, pain threshold at pressure, pain vigilance, and quality of life in patients undergoing orthodontic treatment

**DOI:** 10.1590/1678-2019-0407

**Published:** 2020-03-27

**Authors:** Naila Aparecida Godoi MACHADO, Yuri Martins COSTA, Henrique Muller QUEVEDO, Juliana STUGINSKI-BARBOSA, Caio Martins VALLE, Leonardo Rigoldi BONJARDIM, Daniela Gamba GARIB, Paulo César Rodrigues CONTI

**Affiliations:** 1 Universidade de São Paulo Faculdade de Odontologia de Bauru Departamento de Prótese e Periodontia BauruSão Paulo Brasil Universidade de São Paulo, Faculdade de Odontologia de Bauru, Departamento de Prótese e Periodontia, Bauru, São Paulo, Brasil.; 2 Universidade de São Paulo Grupo de Dor Orofacial de Bauru BauruSão Paulo Brasil Universidade de São Paulo, Grupo de Dor Orofacial de Bauru, Bauru, São Paulo, Brasil.; 3 Universidade de São Paulo Faculdade de Odontologia de Bauru Departamento de Ciências Biológicas BauruSão Paulo Brasil Universidade de São Paulo, Faculdade de Odontologia de Bauru, Departamento de Ciências Biológicas, Seção de Fisiologia da Cabeça e da Face, Bauru, São Paulo, Brasil.; 4 Universidade de São Paulo Faculdade de Odontologia de Bauru Departamento de Odontopediatria, Ortodontia e Saúde Coletiva BauruSão Paulo Brasil Universidade de São Paulo, Faculdade de Odontologia de Bauru, Departamento de Odontopediatria, Ortodontia e Saúde Coletiva, Seção de Ortodontia, Bauru, São Paulo, Brasil.

**Keywords:** Orthodontics, Bruxism, Anxiety, Depression, Quality of life

## Abstract

This study aimed to evaluate whether the presence of awake bruxism was associated with temporomandibular dysfunction symptoms, pain threshold at pressure, pain vigilance, oral health-related quality of life (OHRQoL), and anxiety and depression symptoms in patients undergoing orthodontic treatment.

**Methodology:**

This observational study followed patients who had started receiving orthodontic treatment for six months. The following variables were measured three times (at baseline, one month, and six months): pressure pain threshold (PPT) in the right and left masseter, anterior temporalis, and temporomandibular joint (TMJ), and right forearm; pain vigilance and awareness questionnaire; and shortened form of the oral health impact profile (OHIP-14). Anxiety and depression symptoms were measured using the Beck anxiety inventory and the Beck depression inventory, respectively. The patients were divided into two main groups according to the presence (n=56) and absence (n=58) of possible awake bruxism. The multi-way analysis of variance (ANOVA) was applied on the date (p=0.050).

**Results:**

TMJ and/or muscle pain were not observed in both groups. Time, sex, age group, and awake bruxism did not affect the PPT in the masticatory muscles and pain vigilance (p>0.050). However, the primary effect of awake bruxism was observed when anxiety (ANOVA: F=8.61, p=0.004) and depression (ANOVA: F=6.48, p=0.012) levels were higher and the OHRQoL was lower (ANOVA: F=8.61, p=0.004).

**Conclusion:**

The patients with self-reported awake bruxism undergoing an orthodontic treatment did not develop TMJ/masticatory muscle pain. The self-reported awake bruxism is associated with higher anxiety and depression levels and a poorer OHRQoL in patients during the orthodontic treatment.

## Introduction

Bruxism is frequently implied as a source of microtrauma in the temporomandibular joints (TMJs) and in the mastication muscles. However, the evolution of new definitions and diagnostic criteria for bruxism has great repercussions for the possible relationship between bruxism and craniofacial pain.^1^ An international consensus recently defined bruxism as a repetitive jaw-muscle activity characterized by clenching or grinding of the teeth and/or by bracing or thrusting of the mandible, occurring within two distinct circadian manifestations: sleep and awake bruxism.^1^ Such specifications of the different motor activities and physiological brain states featuring the bruxism manifestations highlight the need to consider their possible different causes and clinical consequences.

Awake bruxism is a masticatory muscle activity during wakefulness that is characterized by repetitive or sustained tooth contact and/or by bracing or thrusting of the mandible, and is not a movement disorder in otherwise healthy individuals.^1^Furthermore, the updated international consensus proposed a bruxism grading system to determine whether a certain bruxism assessment method actually offers a credible outcome. In addition, methods often used in the classification system for the bruxism diagnosis, such as self-report and clinical inspection, have been indicated as some of the only best leads to diagnose probable sleep or awake bruxism, and instrumental approaches are required for definitive bruxism assessments.^1,2^

Until now, the possible relationship between bruxism and symptoms of temporomandibular disorders is still controversial in the literature due to the complexity of etiology and diagnostic of both disorders.^3-5^ The hypothesis often discussed is the possible positive relationship between either awake or sleep bruxism and craniofacial pain is still a commonly held view in the clinical practice^3-5^, and sometimes even presented as a real and simple cause/effect relationship. In line with the perspective that pain-related temporomandibular disorders (TMD) must be envisaged within a biopsychosocial model of illness, and efforts to understand painful temporomandibular disorders along with other chronic pain conditions in a biopsychosocial context have been made.^5-8^ This implies that the association between bruxism and painful temporomandibular disorders has become much more complex.^5^

Orthodontists should be aware of the presence of general and awake bruxism in particular and their possible implications during an orthodontic treatment, such as the excessive use of the jaw and possible association with dental structure (e.g., dental wear and restoration failures), TMJ, and masticatory muscle damage.^9^ Therefore, this study evaluates whether the presence of awake bruxism was associated with the occurrence of temporomandibular dysfunction symptoms, pain threshold at pressure, pain vigilance, oral health-related quality of life (OHRQoL), and anxiety and depression symptoms in patients undergoing orthodontic treatment. This study hypothesized a priori that patients with awake bruxism would present differences in deep pain sensitivity, pain vigilance, anxiety and depression symptoms, and OHRQoL, when compared with those without awake bruxism.

## Methodology

### Sample and ethics

The ethical approval was obtained from the Human Research Ethics Committee of the University of São Paulo, Brazil (CAAE - 09435812.4.0000.5417, June 2016). The participants were informed about the examination procedures and assured of the confidentiality of the collected information. Finally, all participants signed an informed consent form before their inclusion in this study.

The participants’ recruitment was performed by selecting all the patients who started orthodontic treatment with fixed devices in the period from October 2013 to December 2015 in different specialization post-graduate programs in orthodontics. The study was conducted by three examiners who underwent training and calibration to ensure greater reliability for the data collected at different moments of the study. The minimum desired sample size for this study was calculated using an odds ratio (OR) of 2.0; the test power was 70.0% (β= .10), and the standard error was 5% (α= .05).

This observational study followed patients who had started orthodontic treatment with fixed appliance for six months. The patients receiving treatment using removable appliances were not included in the sample. Initially, 162 patients were assessed for eligibility in several orthodontic specialization courses.

The inclusion criteria were the following: a) age between 13 and 60 years; b) absence of temporomandibular disorder (TMD) pain, which was assessed through the third question of the Research Diagnostic Criteria for Temporomandibular Disorders (RDC/TMD) questionnaire: “Have you felt pain on the face, in places such as the cheek (jaw) region, by the side of the head, in front of the ear or in the ear, in the last 4 weeks?”. ^6^ In addition, the exclusion criteria were the following: a) the presence of chronic orofacial pain, such as TMD or primary headache disorders; b) frequent cervical pain, fibromyalgia, and congenital or developmental disorders (e.g., aplasia, hypoplasia, hyperplasia, dysplasia, neoplasia). A detailed medical interview/anamnesis and a clinical examination were performed to fulfill the inclusion and exclusion criteria. No further tests were performed, such as imaging or blood tests.

### Variables

The following variables were measured: a) pressure pain threshold (PPT); b) pain vigilance and awareness questionnaire (PVAQ);^10,11^ c) Beck anxiety inventory (BAI);^12^ d) Beck depression inventory (BDI);^12^ and e) shortened form of the oral health impact profile (OHIP-14).^13^

### PPT

The PPT of right and left masseter, anterior temporalis and temporomandibular joint (TMJ), and right forearm was measured using a digital algometer (KRATOS^®^, Cotia, São Paulo, Brazil). Measurements were obtained with the patient sitting comfortably in an upright position while the examiner pressed the 1-cm^2^ tip at a rate of approximately 0.5 Kg/cm^2^/s. The patients were instructed to press the stop button at the first painful sensation. It was highlighted that the purpose was to measure the minimal amount of pressure at the first perception of pain, and not the pain tolerance. The PPT was determined as the arithmetic mean of two measurements and the average of right and left sides were considered for the statistical analysis (see *Statistics*).

### PVAQ

This self-report questionnaire comprises 16 items and is used to measure the attention to pain. The items are rated on a 6-point scale ranging from 0 (never) to 5 (always) and the final score is the sum of all individual items. The psychometric properties of the original version have shown acceptable values for retention (corrected item-total score correlations ranging from 0.36 to 0.76) and reliability (Cronbach’s alpha = 0.92).^11,14^

### BAI

The anxiety symptoms were measured using the Beck Anxiety Inventory (BAI). This self-report questionnaire comprises 21 items and is used to measure the severity of anxiety. The items are rated on a 4-point scale, ranging from 0 (not at all) to 3 (severely), and the final score is the sum of all individual items. Accordingly, the BAI scores range from 0 to 63, where the higher scores indicated higher anxiety symptoms. This study used the validated version for Portuguese, which showed good psychometrics (Cronbach’s alpha = 0.81).^12^

### BDI

The depression symptoms were measured using the Beck Anxiety Inventory (BAI). This self-report questionnaire comprises 21 items and is used to measure the severity of depression. The items are rated on a 4-point scale, ranging from 0 (not at all) to 3 (severely) and the final score is the sum of all individual items. Accordingly, the BDI-II scores range from 0 to 63, where the higher scores indicated higher depression symptoms. This study used the validated version for Brazilian Portuguese, which showed good psychometrics (Cronbach’s alpha = 0.93).^12^

### OHIP-14

This self-report questionnaire consists of 14 questions divided into seven domains: functional limitation, physical pain, psychological discomfort, physical disability, psychological disability, social disability, and handicap. The items are rated on a 5-point scale, ranging from 0 (never) to 4 (always), and the final score is the sum of all individual items. Accordingly, the OHIP-14 scores range from 0 to 56, where the higher scores indicated poor quality of life related to oral health. This study used the validated version for Brazilian Portuguese, which showed good psychometrics (intraclass coefficient = 0.87 and Cronbach’s alpha = 0.91).^15^

### Design

After the enrollment, the patients were divided into two main groups according to the presence/absence of self-reported awake bruxism. This information was obtained from questions about daytime grinding or clenching of the teeth, which were adapted from the Oral Behavior Checklist (OBC);^16^ nonetheless, this study has not applied the full questionnaire. The patients’ group with awake bruxism was composed according to the answers regularly, often, or always. The patients’ group without awake bruxism was composed according to the following answers: “never”, “sometimes”.

For the age control, the sample was also divided into two groups according to the guidelines of the World Health Organization (WHO) for age groups:^17^ adolescents (aged 10 to 19 years inclusive) and adults (older than 19 years of age).

The variables were assessed at three time-points: T0 = baseline, i.e., just before the moment of installation of the fixed apparatus; T1 = one-month after the installation of the fixed apparatus; and T2 = six-months after the installation of the fixed apparatus.

### Statistics

Quantitative data (age, PPT, PVAQ, BAI, BDI, and OHIP-14) were presented as means and standard deviation (SD) along with the sex distribution. Data normality was assessed using the Kolmogorov-Smirnov test. A log10 transformation was performed when the test results were significant, considering an alpha level of 5% (p<0.050).

A five-way Analysis of Variance (ANOVA) was applied as following: the repeated factor time (3 levels) and the factors site (4 levels), age group (2 levels), sex (2 levels), and awake bruxism (2 levels) were computed to compare the PPT values. In addition, a four-way ANOVA was applied as following: the repeated factor time (3 levels) and the factors age group (2 levels), sex (2 levels), and awake bruxism (2 levels) were computed to compare PVAQ, BAI, BDI, and OHIP-14 values. When appropriate, *post hoc* analyses were performed using the Tukey’s Honestly Statistical Difference (HSD). The significance level was set at 5% (p=0.050).

## Results

One hundred and fourteen healthy participants fulfilled the eligibility criteria and were enrolled in this study. The mean age (SD) of the sample was 24.7 (11.1) and 52% of them were women. In addition, 49% reported awake bruxism, where the mean age (SD) was 27.3 (12.5) and 64% of them were women. The remainder 51% did not report awake bruxism, their mean age (SD) was 22.2 (9.1) and 41% of them were women. Tables 1 and 2 show the PPT values and the psychosocial outcomes throughout the follow-up according to the presence/absence of awake bruxism.

**Table 1:** Mean and standard deviation (SD) of the pressure pain threshold (PPT) of masseter, anterior temporalis, temporomandibular joint (TMJ), and right forearm throughout the study follow-up (p<0.050)

	Baseline	One-month	Six-months
**Awake Bruxism (n=56)**			
Masseter	2.79 (0.84)	2.73 (0.91)	2.81 (0.93)
Anterior Temporalis	2.81 (0.84)	2.77 (0.88)	2.84 (0.83)
TMJ	3.07 (1.07)	3.09 (1.14)	3.10 (1.10)
Right forearm	5.20 (1.72)	5.40 (1.62)	5.36 (1.49)
**W/o Awake Bruxism (n=58)**			
Masseter	2.75 (0.78)	2.88 (0.76)	2.86 (0.76)
Anterior Temporalis	2.83 (0.72)	2.87 (0.72)	2.80 (0.74)
TMJ	3.07 (0.92)	3.21 (0.92)	3.22 (0.90)
Right forearm	5.28 (1.44)	5.29 (1.27)	5.27 (1.20)

**Table 2:** Mean and standard deviation (SD) of the pain vigilance and awareness questionnaire (PVAQ), beck anxiety inventory (BAI), beck depression inventory (BDI), and short-form of the oral health impact profile (OHIP-14) throughout the study follow-up (p=0.050)

	Baseline	One-month	Six-months
**Awake Bruxism (n=56)**			
PVAQ	39.92 (15.20)	38.25 (15.03)	38.00 (14.97)
BAI	8.67 (6.39)	8.74 (6.30)	8.76 (5.93)
BDI	10.32 (8.69)	9.76 (8.33)	9.69 (5.25)
OHIP-14	11.78 (6.18)	10.16 (5.15)	9.71 (5.18)
**W/o Awake Bruxism (n=58)**			
PVAQ	35.86 (16.60)	34.63 (15.70)	33.79 (15.39)
BAI	5.51 (5.18)	5.68 (7.46)	6.10 (7.39)
BDI	7.41 (7.25)	6.82 (7.26)	6.65 (6.41)
OHIP-14	7.94 (7.17)	8.55 (6.20)	8.13 (5.83)

No main effects of time, sex, age group, and awake bruxism were observed on the PPT of masticatory muscles. However, site had a main effect (ANOVA: F=733.64, p<0.001), where the AT and the forearm showed higher thresholds than the masseter and the TMJ (Tukey: p<0.001). Also, there was an interaction between time and sex (ANOVA: F=7.86, p<0.001) where the women’s thresholds at T0 were lower than T2 values (Tukey: p=0.005) and men’s thresholds at T0 (Tukey: p=0.032).

There were no main effects of time, sex and age group, or awake bruxism on the pain vigilance (p>0.050). Also, there were no main effects of time, sex, and age group on the anxiety and depression symptoms. However, there was a main effect of awake bruxism where its presence was related with higher anxiety (ANOVA: F=8.61, p=0.004) and depression (ANOVA: F=6.48, p=0.012) levels. Finally, although there were no main effects of time, sex, and age group on the ORHQoL (p>0.050), a worse ORHQoL was reported by patients with awake bruxism (ANOVA: F=8.61, p=0.004).

## Discussion

Patients with self-reported awake bruxism undergoing orthodontic treatment did not develop TMJ/masticatory muscle pain. The exclusion of patients affected by previous TMJ pain might have biased the study sample. That might have influenced the composition of an artificially “healthier” sample, and, as a possible consequence, decrease the association “strength” between the presence awake bruxism and the pain-related symptoms.

This observational study evaluated the impact of awake bruxism on deep pain sensitivity, pain vigilance, anxiety and depression symptoms, and OHRQoL in patients during orthodontic treatment. The main results were: a) self-reported awake bruxism and six months of orthodontic treatment do not significantly influence the PPT of masticatory muscles in patients without signs and symptoms of TMD pain; b) higher anxiety and depression levels and a worse OHRQoL are found in patients who report awake bruxism regardless of the orthodontic treatment follow-up and age group.

The role of bruxism in the masticatory muscle sensitivity has been questioned.^18,19^ Although it is not possible to make definitive conclusions about the relationship between the presence of awake bruxism and orthodontic treatment, the results of this study showed that the orthodontic treatment and the presence of awake bruxism did not trigger the appearance of signs and symptoms of TMD pain, as no patients have complained of painful TMD after 6 months. These findings agree with the evidence that orthodontic treatment does not necessarily increase the risk of developing TMD.^20^

The results of no influence of neither the self-reported awake bruxism nor the orthodontic treatment on deep pain sensitivity also agree with experimental data, where healthy subjects demonstrated only low levels of pain and fatigue after an experimental tooth clenching paradigm, which was not associated with an altered release of serotonin, glutamate, lactate, or pyruvate.^21^ This evidence weakens the hypothesis that repetitive effort of the masticatory muscles motivated by bruxism episodes would be a risk factor for the development and maintenance of persistent muscle pain. The so called vicious-cycle theory, which was proposed to elucidate the relationship between bruxism episodes and the severity of pain, has not yet demonstrated convincing and definitive acceptance.^22^

Recently it was suggested that individuals with a high degree of oral parafunctional behavior, such as awake bruxism, have some increased occlusal sensitivity.^23^ Michelotti, et al.^24^ (2012) demonstrated that the effects of an experimental occlusal interference differ between individuals reporting a high or low frequency of wake-time oral parafunctions. The interference caused more occlusal discomfort in the high frequency of wake-time oral parafunctions group (HFP) than in the low frequency of wake-time oral parafunctions group and was associated with a significant increase in masticatory muscle pain and headache only in the HFP group. In this study, occlusal alterations resulting from the initial stages of orthodontic treatment associated with self-reported awake bruxism did not contribute to the appearance of symptoms of temporomandibular dysfunction during orthodontic treatment. Furthermore, the presence of awake bruxism was not associated with vigilance pain. New studies may be conducted evaluating the processes of attention in orthodontics, such as somatosensory amplification and hypervigilance.^24^ Clinical experience suggests that individuals with bodily hypervigilance also present with occlusal hypervigilance and continuously check their occlusion.^25^ The reaction to an occlusal alteration resulting from the initial stages of orthodontic treatment may be different in individuals with occlusal hypervigilance, which could justify some patients’ difficulty in adapting to the orthodontic treatment.

The studies involving occlusion, orthodontics, and TMD up to the present moment have focused on the incidence and prevalence of dysfunctions in certain samples, seeking to establish the relative risk of this disease. However, since temporomandibular disorders are complex entities, the relationship between these entities should be seen in the pain models, inserting the biopsychosocial perspective in the evaluations. In the researchers’ understanding, this is the first study to investigate the signs and symptoms of TMD in orthodontic patients, which also approaches the psychosocial aspects in the same sample.

Previous studies have highlighted the need to consider the psychological dimensions before and during the orthodontic treatment.^26-29^ The literature is scarce regarding information assessing anxiety and depression symptoms in patients during orthodontic treatment. Even though the results did not found a significant effect of the orthodontic treatment on psychosocial variables, the impact of awake bruxism on anxiety and depression symptoms and quality of life reinforce previous findings. They have showed an association with psychosocial factors, such as stress, disturbed personality, anxiety; psychopathological factors, such as smoking, consumption of alcohol and caffeine; and genetic factors.^30,31^ Traditional assumptions that peripheral factors such as occlusal discrepancies and deviations of the facial anatomy would be possible causes of bruxism have been discredited.^32^

Clinicians should be aware of the patients’ psychological traits before starting orthodontic treatment, because these traits may be related to the presence of awake bruxism and to a significant impact on quality of life, compromising the patient’s adaptation during orthodontic treatment. In addition, previous identification of patients with awake bruxism may have implications for the design of the treatment plans on measures, such as cognitive behavioral approaches that can be included and help patients understand their need for relaxed mastication muscles maintenance. This study has some limitations: a) the last international consensus defined and graded sleep and awake bruxism and determined that the self-reported diagnosis is classified as possible bruxism. In this study, the diagnosis of bruxism was performed without any clinical examination, which represents a lower grade of bruxism diagnosis uncertainty (“possible”) according to the international bruxism group.^1^ However, it is also important to note that self-reported measures of oral parafunction have showed greater prognostic value than clinical assessment;^33^ b) the relatively large range between the follow-ups would have decreased the chance to detect transient changes, in particular the ones related to pain sensitivity, e.g., experimental data has showed that PPT is reduced after placing orthodontic separators.^34^

## Conclusion

The self-reported awake bruxism is associated with higher anxiety and depression symptoms and poorer OHRQoL in patients during orthodontic treatment. On the other hand, short-term orthodontic treatment does not impact on deep pain sensitivity, pain vigilance, degree of anxiety and depression, and OHRQoL.
